# Pharmacological Inhibition of Cyclin-Dependent Kinases Triggers Anti-Fibrotic Effects in Hepatic Stellate Cells In Vitro

**DOI:** 10.3390/ijms21093267

**Published:** 2020-05-05

**Authors:** Anna Hübbers, Julia Hennings, Daniela Lambertz, Ute Haas, Christian Trautwein, Yulia A. Nevzorova, Roland Sonntag, Christian Liedtke

**Affiliations:** 1 Department of Internal Medicine III, University Hospital, RWTH Aachen University, D-52074 Aachen, Germany; ahuebbers@ukaachen.de (A.H.); jhennings@ukaachen.de (J.H.); dlambertz@ukaachen.de (D.L.); uhaas@ukaachen.de (U.H.); ctrautwein@ukaachen.de (C.T.); ynevzorova@ukaachen.de (Y.A.N.); 2 Department of Genetics, Physiology, and Microbiology, Faculty of Biology, Complutense University Madrid, 28040 Madrid, Spain

**Keywords:** hepatic stellate cells, liver fibrosis, cyclin-dependent kinase, CR8, cell cycle, DNA repair

## Abstract

Liver fibrosis is a wound healing process in response to chronic liver injury, which is characterized by the accumulation of extracellular collagen produced by Hepatic Stellate Cells (HSCs). This process involves cell cycle re-entry and proliferation of normally quiescent HSCs controlled by cyclins and associated cyclin-dependent kinases (Cdks). Cdk2 mediates the entry and progression through S-phase in complex with E-and A-type cyclins. We have demonstrated that cyclin E1 is essential for liver fibrogenesis in mice, but it is not known if this is dependent on Cdk2 or related Cdks. Here, we aimed to evaluate the benefit of the pan-Cdk inhibitor CR8 for treatment of liver fibrosis in vitro. CR8-treatment reduced proliferation and survival in immortalized HSC lines and in addition attenuated pro-fibrotic properties in primary murine HSCs. Importantly, primary murine hepatocytes were much more tolerant against the cytotoxic and anti-proliferative effects of CR8. We identified CR8 dosages mediating anti-fibrotic effects in primary HSCs without affecting cell cycle activity and survival in primary hepatocytes. In conclusion, the pharmacological pan-Cdk inhibitor CR8 restricts the pro-fibrotic properties of HSCs, while preserving proliferation and viability of hepatocytes at least in vitro. Therefore, CR8 and related drugs might be beneficial for the treatment of liver fibrosis.

## 1. Introduction

Initiation of liver fibrosis is a consequence of chronic liver injury induced for instance by chronic viral hepatitis, fatty liver disease or excessive alcohol consumption. As a result of these inflammatory processes, part of the liver parenchyma undergoes cell death and is subsequently replaced by scar tissue through the accumulation of extracellular matrix (ECM) proteins [1]. Especially, two cellular events take place during initiation and progression of liver fibrosis, which are in part independent of each other. First, hepatocyte death triggers compensatory proliferation of remnant hepatocytes in order to maintain liver homeostasis [2,3]. Second, liver injury stimulates the activation of resting, vitamin A-storing Hepatic Stellate Cells (HSCs) into proliferating, activated and ECM-producing myofibroblasts [4]. The latter ones are further characterized by expression of alpha smooth muscle actin (αSMA) and collagens in the liver [5].

The transition from quiescence into a proliferating state is typically controlled by specific complexes of cyclins (Ccn) and cyclin-dependent kinases (Cdks). E-type cyclins (CcnE1, CcnE2) together with the catalytic subunit Cdk2 usually regulate the onset of DNA-synthesis referred to as S-phase [6]. Importantly, we have recently described a unique role of CcnE1 for activation, proliferation and survival of HSCs. Accordingly, knockout mice constitutively lacking CcnE1 were protected from carbon tetrachloride (CCl_4_)-induced liver fibrosis in vivo [7]. In addition, we already demonstrated that delivery of small inhibitory RNAs (siRNA) directed against CcnE1 can prevent both the onset of liver fibrosis and the progression of already existing fibrosis in mice [3]. Thus, CcnE1 seems to be a suitable target for the treatment of liver fibrosis. However, so far it is completely unknown if the pro-fibrotic function of CcnE1 depends on its canonical interaction partner Cdk2.

Cdks are catalytically active cell cycle kinases and thus represent promising targets for small inhibitory molecules that modulate cell proliferation. Chemical inhibition of Cdk2 is based on the interaction between the adenosine triphosphate (ATP) binding pocket and a modified adenine derivate [8]. The small-molecule inhibitor (R)-Roscovitine was one of the first inhibitors described with strong specificity for Cdk2, but also for Cdk1, 5, 7 and 9 among others [9]. (R)-Roscovitine has been tested in clinical trials for non-small cell lung cancer, B cell malignancies, thymic carcinoma and breast cancer, and has shown limited toxicity and moderate side effects [10,11,12]. However, the benefit of roscovitine for clinical purposes was limited by factors such as short half-life and rather poor potencies on Cdk inhibition and tumor cells. More recently, an improved roscovitine-derivative has been development referred to as CR8. CR8 is much more potent in selectively inhibiting Cdk 1, 2, 5, 7 and 9 and in induction of apoptotic cell death when compared to roscovitine [13,14,15,16].

In the present study we wanted to investigate the effectiveness of CR8 for the inhibition of cell cycle activity of immortalized HSC lines and primary HSCs. Of note, our designed anti-fibrotic approach should not affect the vitality of hepatocytes, as this is required for liver homeostasis. We will show that CR8 treatment at relatively low dosages can lead to a selective inhibition of proliferation, activation and survival of HSCs without considerably affecting the viability and proliferation capacity of hepatocytes in vitro. Thus our study defines the use of small-molecule pan-Cdk inhibitors as a potential new strategy to treat liver fibrosis.

## 2. Results

### 2.1. CR8 Induces Apoptosis in Immortalized Murine and Human HSC Lines

**Figure 1:**
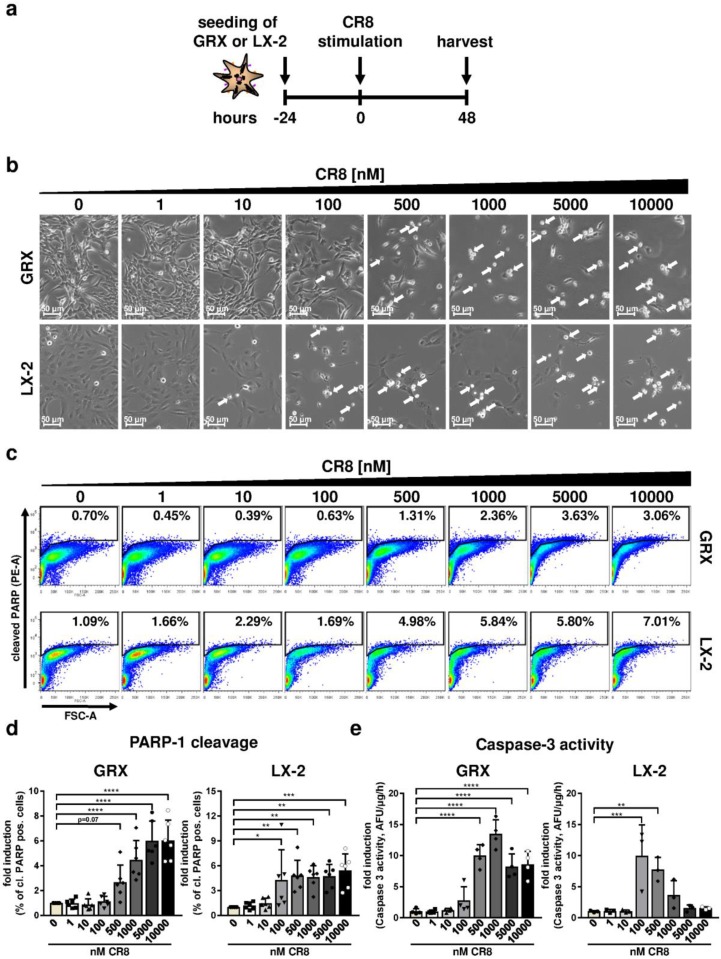
CR8-treatment triggers apoptosis in murine and human hepatic stellate cell lines. Murine GRX and human LX-2 cells were treated for 48 h with increasing concentrations of CR8 as indicated. Dimethyl sulfoxide (DMSO) treatment alone (0 nM) served as control. (**a**) Schematic overview of the experimental design. (**b**) GRX (upper panels) and LX-2 (lower panels) cells were investigated by brightfield microscopy for cell density and morphology at a magnification of 100×. Arrows highlight round-shaped cells, indicative for cell death. (**c**,**d**) Cells were stained with fluorescence-labelled antibodies against cleaved poly (ADP-ribose)-polymerase 1 (PARP-1) and analyzed via flow cytometry, in particular fluorescence-associated cell sorting (FACS). (**c**) Representative FACS-plots of cleaved PARP-1 positive (% of single cells) GRX (upper panels) and LX-2 (lower panels) cells after treatment with increasing concentrations of CR8. (**d**) Quantification of cleaved PARP-1 positive GRX (left panel) and LX-2 (right panel) cells (% of single cells) from *n* = 6 independent FACS experiments. Data are shown as fold induction compared to controls. (**e**) Specific caspase-3 enzyme activity in GRX (left panel, *n* = 4) and LX-2 (right panel, *n* = 3) cells after CR8 treatment. Values are given as arbitrary fluorescence units (AFU)/μg protein/h and are calculated as fold induction in comparison to controls. Data reflect the mean of at least *n* = 3 independent experiments, unless otherwise indicated. * *p* < 0.05; ** *p* < 0.01; *** *p* < 0.001, **** *p* < 0.0001.

**Figure A1:**
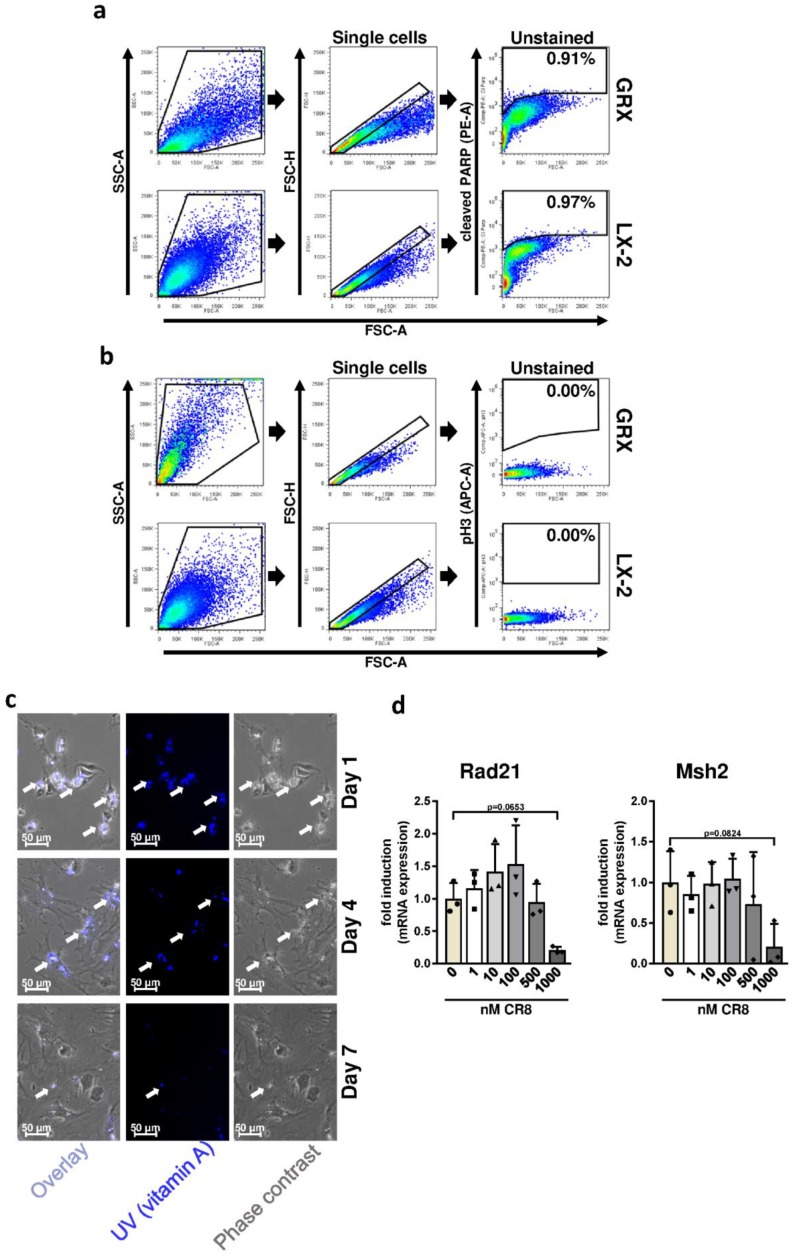
Supplementary data related to Figure 1, Figure 3 and Figure 5. (**a**,**b**) Murine GRX and human LX-2 cells were treated for 48 h with increasing concentrations of CR8 as indicated. Dimethyl sulfoxide (DMSO) treatment (0 nM) served as control. Gating strategy for identification and quantification of cleaved cleaved poly (ADP-ribose)-polymerase 1 (PARP-1) (**a**) or phosphorylation of histone H3 (pH3)-positive (**b**). GRX (upper panels) and LX-2 (lower panels) cell populations via flow cytometry (FACS) analysis. Left panel: Forward versus side scatter (FSC vs. SSC) gating for identification of cells of interest. FSC indicates cell size, SSC indicates cell granularity. Middle panel: FSC-A vs. FCS-H gating for identification of single cells. Right panel: Unstained control, which was not incubated with antibodies to assess background. (**c**,**d**) Primary murine hepatic stellate cells (HSCs) were isolated from livers of 20–30-week-old wildtype mice, cultured for 7 days and subsequently treated with increasing concentrations of CR8 for 48 h. Dimethyl sulfoxide (DMSO) treatment alone (0 nM) served as control. (**c**) Cell morphology (phase contrast) and vitamin A storage (UV) of the cells was controlled over indicated time via brightfield and fluorescence microscopy, confirming proper transdifferentiation of HSCs towards activated myofibroblasts by loss of vitamin A. White arrows indicate vitamin A-positive HSCs. (d) RAD21 and MutS homolog 2 (MSH2) gene expression in HSCs (*n* = 3) were determined by qPCR. Data were normalized against the expression of glyceraldehyde-3-phosphate dehydrogenase and calculated as fold induction compared to controls. *p*-values are indicated in panels.

In order to evaluate basic effects of CR8 on HSCs, we initially took advantage of the well-established, immortalized and permanently proliferating HSC lines GRX (murine origin) and LX-2 (human origin). These cell lines were treated with increasing concentrations of CR8 (1-nM) for 48 h and subsequently subjected to analysis for cell density, morphology and markers of apoptosis (Figure 1a). Following CR8-treatment, both cell lines showed a dose-dependent reduction of cell density and an enrichment of round-shaped cells. Of note, these effects were observed at CR8 concentrations ≥ 500 nM in murine GRX cells, but already at concentrations ≥ 100 nM in human LX-2 cells (Figure 1b). Hence, these observations indicate anti-proliferative and cell death-inducing effects of CR8 on HSC lines. In order to identify the mode of cell death in these cells, we measured cleavage of PARP-1 (poly (ADP-ribose)-polymerase 1), which is an established marker of apoptosis [17]. Fluorescence-associated cell sorting (FACS) of CR8-treated GRX and LX-2 cells revealed a dose-dependent increase of cleaved PARP-1 (i.e., apoptosis) in both cell lines (Figure 1c,d). The gating strategy for quantification of cleaved PARP-1 is shown in Figure A1a. To confirm that CR8 induces apoptosis in GRX and LX-2 cells, we measured enzymatic caspase-3 activity in these cells as described earlier [18]. CR8 substantially induced caspase-3 activation up to 10-fold in both HSC lines (Figure 1e). As observed before, GRX cells turned out to be less sensitive to CR8 than LX-2 cells with regard to caspase-3 activation (compare Figure 1e left versus right panel). Taken together, CR8 efficiently triggers apoptosis in both murine and human HSC lines.

### 2.2. Pharmacological Inhibition of Cdks Limits Cell Cycle Activity and Triggers G2 Arrest in Murine and Human HSC Cell Lines

**Figure 2:**
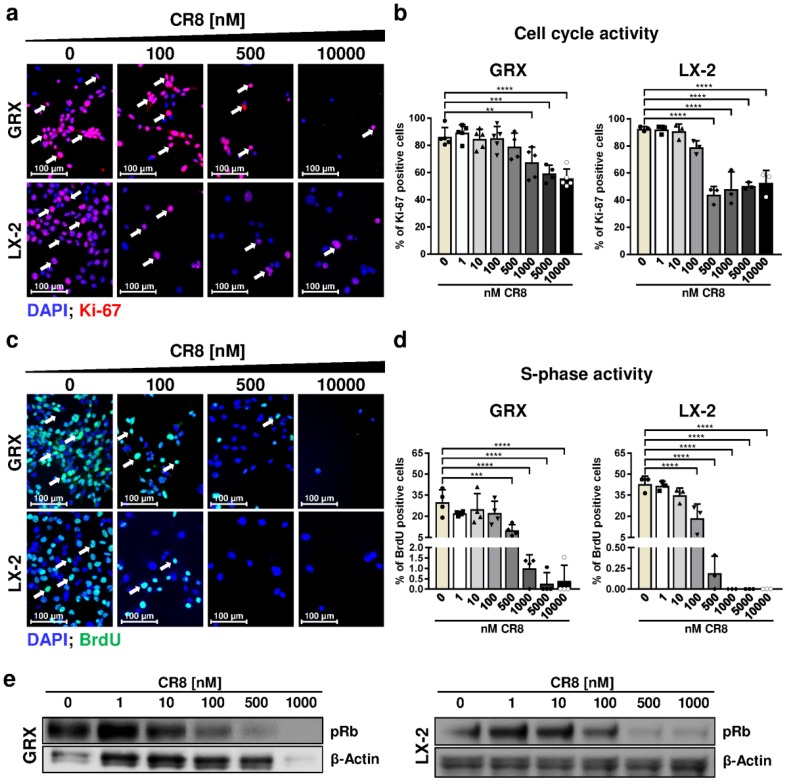
CR8-mediated inhibition of cyclin-dependent kinases (Cdks) reduces cell cycle activity in murine and human hepatic stellate cell lines. GRX and LX-2 cells were treated for 48 h with increasing concentrations of CR8 as indicated. Dimethyl sulfoxide (DMSO) treatment alone (0 nM) served as control. Cells were treated 2 h before harvest with 5-bromo-2′-deoxyuridine (BrdU). (**a**) Representative fluorescence microscopy images of GRX (upper panels) and LX-2 (lower panels) cells after staining with a fluorescence-labelled antibody against Ki-67 (red, arrows). Nuclei were counterstained with 4,6-diamino-2-phenylindole (DAPI, blue). (**b**) Quantification of data shown in (**a**). Ki-67 positive GRX (left panel, *n* = 4) and LX-2 (right panel,) cells from independent experiments were quantified and calculated as percent of total DAPI-positive cells. (**c**) Representative images of GRX (upper panels) and LX-2 (lower panels) cells after staining with a fluorescence-labelled antibody against BrdU (green, arrows). Nuclei were counterstained with DAPI (blue). (**d**) Percentage of BrdU-positive GRX (left panel, *n* = 4) and LX-2 (right panel,) cells. Data reflect the mean from independent experiments. (**e**) Immunoblot analysis for phosphorylated retinoblastoma protein (pRb) in GRX (left panel) and LX-2 (right panel) cells. β-Actin expression was determined as internal loading control. Please note that β-Actin expression is also regulated by high CR8 concentrations. Values are means of at least *n* = 3 independent experiments, unless indicated otherwise. ** *p* < 0.01; *** *p* < 0.001, **** *p* < 0.0001.

As we showed that CR8 dose-dependently reduces cell density and efficiently induces intrinsic apoptosis, we now investigated if Cdk inhibition by CR8 acts anti-proliferative in continuously proliferating and activated murine and human HSC cell lines. Therefore, the general cell cycle activity was analyzed by immunofluorescence staining of the proliferation marker Ki-67. The amount of double positive DAPI/Ki-67 cells were significantly reduced with increasing CR8 concentrations. We found that murine GRX cells exhibited a 10% reduction of proliferation at concentration ≥ 1000 nM with a maximum reduction of approximately 20% at the highest concentration tested (nM). In comparison, proliferation of LX-2 cells was already significantly decreased at a CR8 concentration of 500 nM with a strong reduction of about 50% of the Ki-67-positive cells (Figure 2a,b). Next, we performed a more detailed cell cycle analysis by performing 5-bromo-2′-deoxyuridine (BrdU) incorporation experiments in order to identify cells in S-phase. CR8 dose-dependently reduced the number of cells in S-phase in both murine GRX and human LX-2 cells with different efficiency. In LX-2 cells, a concentration of 100 nM CR8 was sufficient to significantly impair S-phase, whereas in GRX cells a minimum of 500 nM CR8 was required to obtain first inhibitory effects (Figure 2c,d).

The potential of CR8 for the inhibition of Cdk2 kinase activity and S-phase was further investigated by analysis of retinoblastoma protein (Rb) phosphorylation in GRX and LX-2 cells. Rb is a canonical phospho-target of Cdk2 during S-phase initiation, and impaired Rb phosphorylation (pRb) after CR8-treatment thus proves inhibition of Cdk activity [3]. Immunoblot analysis revealed that CR8-treatment impaired Rb phosphorylation in both GRX and LX-2 cells in a dose-dependent manner (Figure 2e). Of note, CR8 also affected the expression of the internal loading control β-Actin at high dose (1000 nM) in murine cells, which could be potentially due to the known effects of CR8 on inhibition of the transcriptional co-factors Cdk7 and Cdk9 [13].

**Figure 3:**
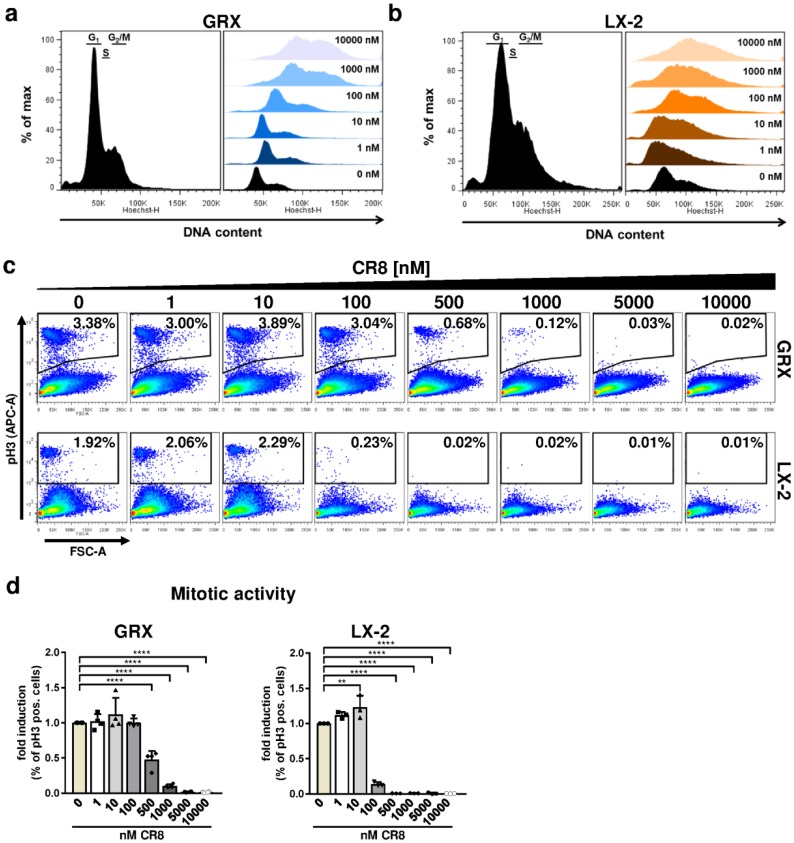
CR8-treatment triggers accumulation of immortalized hepatic stellate cells in mitosis. GRX and LX-2 cells were treated for 48 h with increasing concentrations of CR8. Dimethyl sulfoxide (DMSO) treatment alone (0 nM) served as control. (**a**,**b**) Determination of cellular DNA content in (**a**) murine GRX and (**b**) human LX-2 cells through DAPI staining and flow cytometry (FACS) analysis. Left panels: histograms showing DNA content and the corresponding assignment to the distinct cell cycle phases in untreated (**a**) murine GRX and (**b**) human LX-2 cells as follows: G1: 2n DNA content, S: 2-4n; G2/M: 4n. Right panels: DNA content distribution of CR8-treated (**a**) murine GRX and (**b**) human LX-2 cells at indicated concentrations. (**c**,**d**) Cells were stained with an antibody directed against phosphorylated Histone H3 (pH3) and analyzed by FACS. (**c**) Representative FACS-plots of cleaved pH3-positive (% of single cells) GRX (upper panels) and LX-2 (lower panels) cells at different concentrations of CR8. (**d**) Quantification of pH3-positive GRX (left panel, *n* = 4) and LX-2 (right panel) cells (% of single cells) as fold induction compared to controls by FACS. Values are means of at least *n* = 3 independent experiments, unless otherwise indicated. ** *p* < 0.01; **** *p* < 0.0001.

We next determined the effect of CR8 on the DNA content of both HSC lines by FACS analysis with the aim to assign individual cells to G1, S or G2/M-phase of the cell cycle. The typical DNA distribution of continuously growing but untreated HSCs is shown in the left panel of Figure 3a (murine GRX cells) and Figure 3b (human LX-2 cells). Interestingly, CR8 treatment caused a substantial increase in GRX and LX-2 cells with a high DNA content (i.e., 4n or more) pointing to polyploidization and arrest at the G2-phase (Figure 3a,b, right panels). We therefore determined the phosphorylation of histone H3 (pH3), which is an established marker of mitotic cells. The gating strategy for quantification of pH3-positive cells is shown in Figure A1b. Interestingly, CR8 triggered the decrease of pH3-positive cells in a dose-dependent manner as determined by FACS analysis (Figure 3c,d). In summary, our results point to a CR8-dependent anti-proliferative effect due to Cdk inhibition in murine and human HSCs. The reduced overall cell cycle activity is accompanied by aberrations in DNA-synthesis and an arrest in the G2-phase in both murine and human cell lines although with a different strength of response.

### 2.3. CR8 Dose-Dependently Induces DNA Double-Strand Breaks in Murine and Human HSC Lines

**Figure 4:**
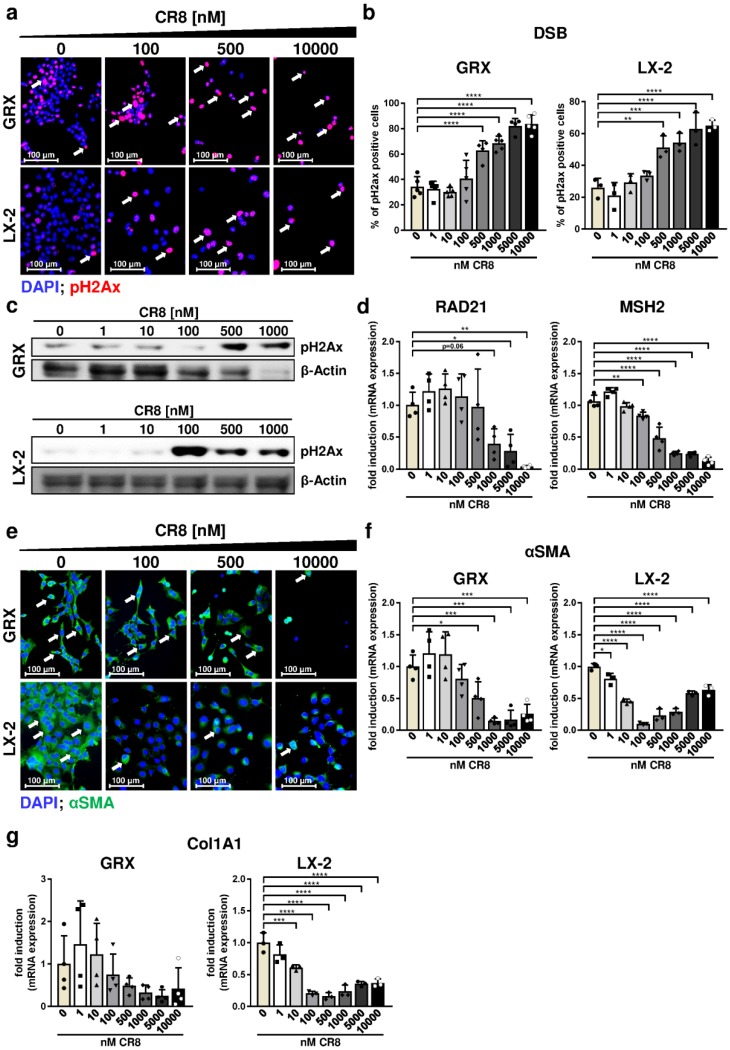
CR8 treatment induces DNA double-strand breaks and reduces pro-fibrotic properties in immortalized HSC lines. Murine GRX and human LX-2 cells were treated for 48 h with increasing concentrations of CR8 as indicated. DMSO treatment (0 nM) served as control. (**a**) Representative fluorescence microscopy images of GRX (upper panels) and LX-2 (lower panels) cells after staining of phosphorylated Histone H2Ax (pH2ax, red, arrows). Nuclei were counterstained with DAPI (blue). (**b**) Quantification of pH2Ax-positive GRX (left panel, *n* = 5) and LX-2 (right panel) cells in % as a measure of DNA double-strand breaks (DSB). (**c**) Detection of pH2Ax by immunoblot analysis in GRX (upper panel) and LX-2 (lower panel) cells. β-Actin expression was determined as internal loading control. (**d**) RAD21 and MutS homolog 2 (MSH2) gene expression in GRX cells (*n* = 4) were determined by qPCR. Data were normalized against the expression of glyceraldehyde-3-phosphate dehydrogenase (GAPDH) and calculated as fold induction compared to controls. (**e**) Representative fluorescence microscopy images of GRX (upper panels, *n* = 4) and LX-2 (lower panels) cells after fluorescence staining of alpha smooth muscle actin (αSMA, green, arrows). Nuclei were counterstained with DAPI (blue). (**f**,**g**) αSMA (**f**) and Collagen 1A1 (Col1A1, (**g**)) expression in GRX (left panel) and LX-2 (right panel) cells was determined by qPCR. Data were normalized against the expression of GAPDH and calculated as fold induction compared to controls. All quantitative values are means of at least *n* = 3 independent experiments, unless otherwise indicated. * *p* < 0.05; ** *p* < 0.01; *** *p* < 0.001, **** *p* < 0.0001.

The previous results indicated that overall impaired cell cycle activity by CR8 is associated with loss of S-phase activity (Figure 2d) and G2-phase arrest (Figure 3a,b). We thus hypothesized that CR8 could affect DNA integrity and/or DNA repair, which could force cells to maintain in a state of incompletely replicated DNA. To address this question, we determined the level of phosphorylated histone H2Ax (pH2Ax), which is a specific marker for DNA double-strand breaks (DSBs) [19], in GRX and LX-2 cells using immunofluorescence microscopy and immunoblot analysis. As expected, CR8 induced significantly increased numbers of pH2Ax-positive cells in both cell lines at concentration ≥ 500 nM (Figure 4a,b). These data were confirmed by western blot analysis (Figure 4c). However, as reported before, high concentrations of CR8 also inhibited expression of the internal loading control β-Actin in GRX cells, which hinders interpretation of this result at least in murine cells. Overall, however, our data indicated that CR8 induces double strand breaks in HSCs in a dose-dependent manner.

We next analyzed whether the induction of DSBs by CR8 was associated with an induction of DNA repair genes. To this end, the gene expression of RAD21 and MSH2 (MutS homolog 2) was investigated. RAD21 is involved in the repair of DSBs as well as in chromatid cohesion during mitosis [20], while MSH2 is part of the DNA mismatch repair machinery [21]. Unexpectedly, gene expression of RAD21 or MSH2 was not induced by any tested CR8 concentration in GRX cells (Figure 4d). On the contrary, expression of both genes was even down-regulated under these conditions suggesting that CR8-treatment is associated with both DNA damage and a lack of DNA damage response.

### 2.4. CR8-Treatment Reduces the Pro-Fibrotic Properties of HSC Lines In Vitro

We next tested if CR8-treatment had an impact on the pro-fibrotic properties of immortalized and thus activated HSC lines. Typically, activated myofibroblasts synthesize large amounts of pro-fibrotic proteins such as collagen type 1 and αSMA. Fluorescence microscopy revealed a dose-dependent reduction of αSMA-positive GRX and LX-2 cells by CR8 (Figure 4e). This finding was confirmed by quantification of αSMA gene expression (Figure 4f). In good agreement, CR8 also inhibited gene expression of the extracellular matrix gene collagen type 1 alpha chain 1 (Col1A1) in LX-2 cells (Figure 4g). Regarding murine GRX cells, the impact of CR8 on Col1A1 expression was unclear due to high variations (Figure 4g). These findings support the idea that CR8 mediates anti-fibrotic effects in HSCs at different levels.

### 2.5. CR8-Mediates Anti-Fibrotic Effects and Triggers DNA Damage in Primary Murine HSCs

So far, we demonstrated that CR8 reduced survival, proliferation and pro-fibrotic properties of continuously proliferating human and murine HSC lines. However, the response of primary cells derived from liver much better reflects the situation in vivo. In addition, for an efficient therapy of liver fibrosis, it would be essential to inhibit HSC activation without impairing the regeneration capacity of healthy hepatocytes. Thus, in the second part of the study we investigated the response of primary murine HSCs and hepatocytes towards CR8 with the aim to define effective CR8 dosages that reduce the pro-fibrotic properties of HSCs without affecting hepatocyte function.

**Figure 5:**
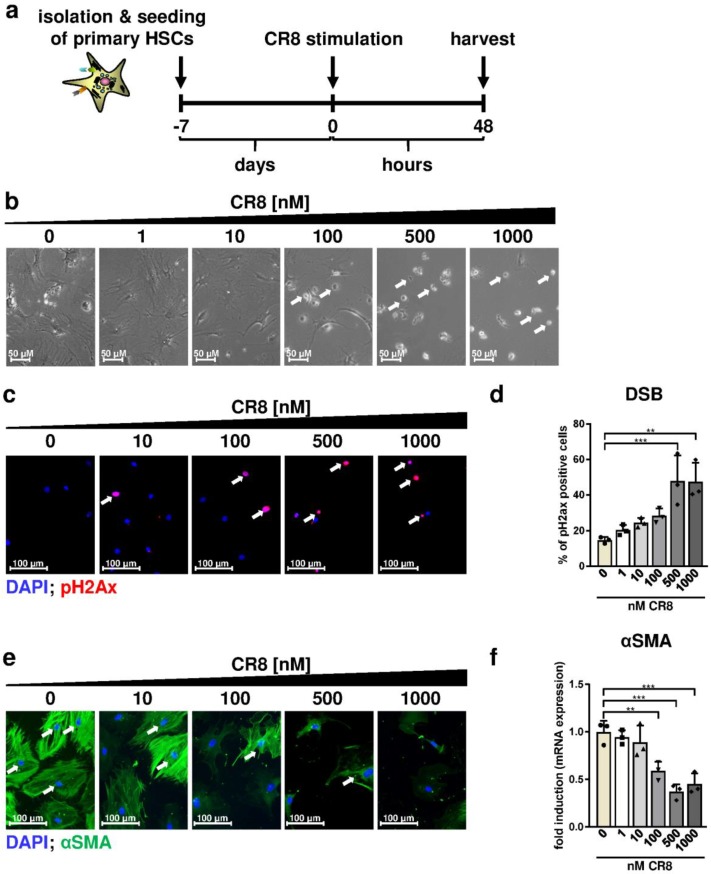
CR8 reduces viability, pro-fibrotic activity and DNA integrity in primary murine HSCs. (**a**) Schematic overview of the experimental design. Primary murine HSCs were isolated from livers of 20–30-week-old wildtype mice, cultured for 7 days and subsequently treated with increasing concentrations of CR8 for 48 h. DMSO treatment alone (0 nM) served as control. (**b**) Brightfield microscopy of HSCs. White arrows indicate round-shaped cells, indicative for cell death. (**c**) Representative fluorescence microscopy images of HSCs after staining of phosphorylated Histone H2Ax (pH2Ax, red, arrows). Nuclei were counterstained with DAPI (blue). (**d**) Quantification of pH2Ax-positive HSCs in % as a measure of DNA double-strand breaks (DSB). (**e**) Representative fluorescence microscopy images of HSCs after staining of alpha smooth muscle actin (αSMA, green, arrows). Nuclei were counterstained with DAPI (blue). (**f**) Normalized gene expression analysis of αSMA by qPCR. Data were normalized against the expression of glyceraldehyde-3-phosphate dehydrogenase and calculated as fold induction compared to controls. All quantitative values are means of *n* = 3 independent experiments. ** *p* < 0.01; *** *p* < 0.001.

In the first step, murine primary HSCs were isolated from wildtype mice, plated and cultured for 7 days to obtain transdifferentiated and activated myofibroblasts. Cell morphology and vitamin A storage of the cells was controlled over time confirming proper transdifferentiation of HSCs towards activated myofibroblasts (Figure A1c). Subsequently, these transdifferentiated cells were treated with increasing concentrations of CR8 for 48 h and analyzed thereafter (Figure 5a). After treatment with CR8 concentrations ≥ 100 nM, a substantial reduction in cell density and the presence of single round-shape cells in the supernatant was observed pointing to reduced proliferation and/or induction of cell death (Figure 5b). Importantly, CR8 concentrations ≥ 500 nM resulted in a substantial and significant increase in DSBs as determined by pH2Ax stainings in primary HSCs (Figure 5c) which is in good agreement with the previous data obtained from HSC lines (compare Figure 4a,b). Of note, accumulation of DSBs in these cells was not associated with induction of DNA repair genes such as RAD21 and MSH2 (Figure 1Ad), which is again consistent with our findings in murine GRX cells (compare Figure 4d). We then investigated if CR8 may also affect the pro-fibrotic properties of primary self-activated HSCs. In fact, CR8 treatment with concentrations ≥ 100 nM significantly reduced protein and gene expression of αSMA, as determined by fluorescence microscopy and qPCR (Figure 5e,f). In summary, CR8 mediated comparable anti-fibrotic and anti-proliferative effects in primary murine HSCs and continuously proliferating HSC lines starting at a critical concentration of 100 nM.

### 2.6. Primary Hepatocytes Are Less Sensitive to CR8 Compared to HSCs with Regard to Cell Survival and DNA Damage Induction

**Figure 6:**
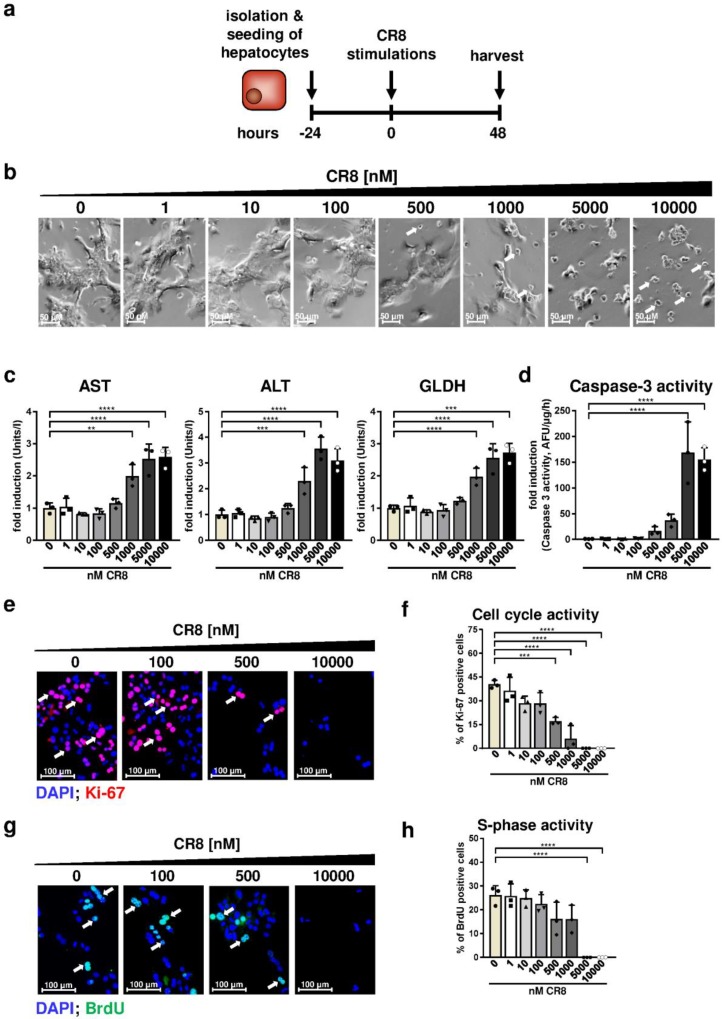
CR8 treatment reduces cell cycle activity of primary murine hepatocytes with moderate effects on viability. (**a**) Schematic overview of the experimental design. Primary hepatocytes were isolated from livers of 10-week-old wildtype mice, plated and treated with increasing concentrations of CR8 for 48 h. DMSO treatment (0 nM) served as control. Cells were treated 2 h before harvest with 5-bromo-2′-deoxyuridine (BrdU). (**b**) Brightfield microscopy of primary hepatocytes. Arrows indicate round-shaped cells, indicative for cell death. (**c**) Activities of aspartate aminotransferase (AST, left panel), alanine aminotransferase (ALT, middle panel) and glutamate dehydrogenase (GLDH, right panel) indicative for hepatocyte death were measured from cell culture supernatant of hepatocytes and are shown as fold induction compared to controls. (**d**) Specific caspase-3 enzyme activity (AFU/µg protein/h) of hepatocytes after CR8 treatment. Values are calculated as fold induction in comparison to controls (*n* = 3). (**e**) Representative fluorescence microscopy images of primary hepatocytes after Ki-67 staining (red, arrows). Nuclei were counterstained with DAPI (blue). (**f**) Quantification of Ki-67-positive cells in %. (**g**) Representative immunofluorescence images of BrdU-stained primary hepatocytes (green, arrows). Nuclei were counterstained with DAPI (blue). (**h**) Quantification of BrdU-positive cells in %. All quantitative values are means of at least *n* = 3 independent experiments, unless otherwise indicated. ** *p* < 0.01; *** *p* < 0.001; **** *p* < 0.0001.

The previous analyses demonstrated inhibitory effects of CR8 on survival and pro-fibrotic activity of primary HSCs at a critical concentration of 100 nM. In subsequent experiments, we aimed to define CR8 concentrations, which are critical for survival, proliferation and DNA integrity of primary hepatocytes. To address this issue, primary hepatocytes were isolated from wildtype mice and stimulated for 48 h with increasing CR8 concentrations exactly as was the case for the treatment of immortalized and primary HSCs (Figure 6a).

Under these conditions, CR8 only caused a reduced cell density, cell shrinking and increasing amounts of round-shaped cells at high concentrations of ≥1000 nM (Figure 6b). AST (aspartate aminotransferase), ALT (alanine aminotransferase) and GLDH (glutamate dehydrogenase) are abundantly expressed in hepatocytes and are non-specifically released during cell death, therefore serving as clinical standard serum markers for liver damage. AST, ALT and GLDH levels were significantly elevated at CR8 concentrations ≥ 1000 nM, pointing to CR8-mediated hepatocyte death (Figure 6c). In good agreement with the found serum marker levels, CR8 concentrations ≥ 5000 nM also resulted in an induction of caspase-3 although, pointing to a pro-apoptotic effect of CR8 on primary hepatocytes at high dosages (Figure 6d). Overall, these data suggest that primary hepatocytes tolerate much higher CR8 doses than primary HSCs.

**Figure 7:**
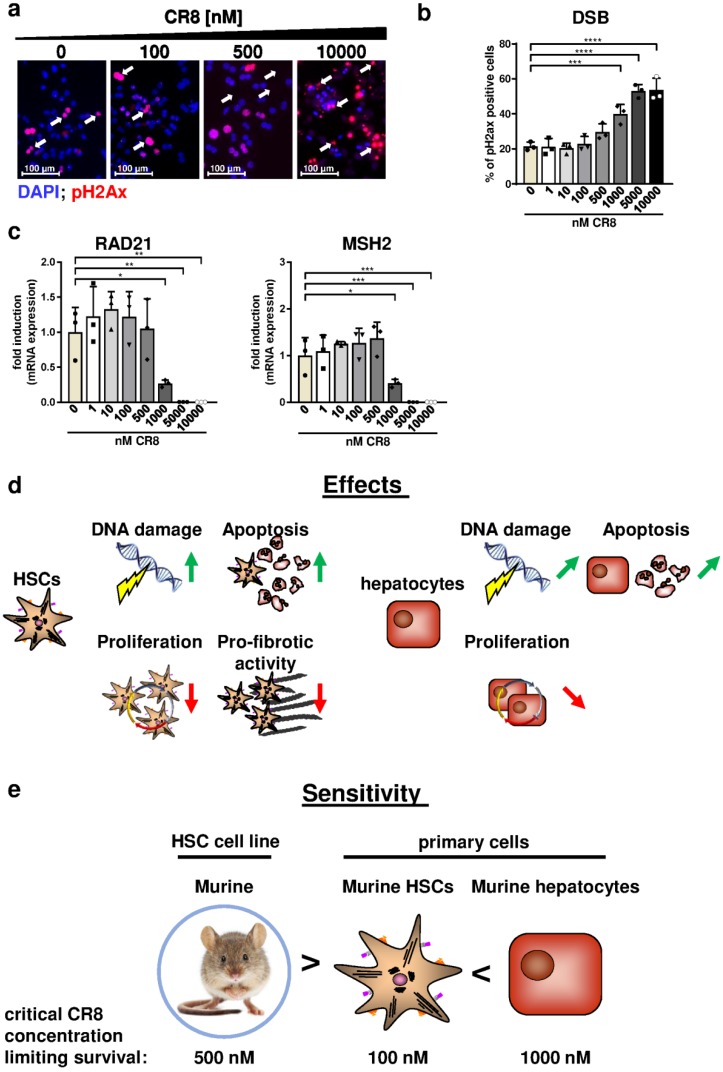
CR8 has a less severe impact on DNA integrity in hepatocytes compared to HSCs. Primary hepatocytes were isolated, cultivated overnight and subsequently treated with increasing concentrations of CR8 for 48 h. DMSO treatment (0 nM) served as control. (**a**) Representative fluorescence images of phosphorylated Histone H2Ax (pH2Ax)-stained primary hepatocytes (red, arrows). Nuclei were counterstained with DAPI (blue). (**b**) Quantification of pH2Ax-positive cells in % as a measure of DNA double-strand breaks (DSB). (**c**) RAD21 (left panel) and MSH2 (right panel) gene expression were determined by qPCR. Data were normalized against of GAPDH expression and shown as fold induction compared to controls. All quantitative values are means of *n* = 3 independent experiments. * *p* < 0.05; ** *p* < 0.01; *** *p* < 0.001.; **** *p* < 0.0001. (**d**) Graphic summary of CR8-mediated effects on HSCs and hepatocytes. In HSCs (primary cells or cell lines, left), CR8 induces DNA damage and apoptosis, but decreases proliferation and pro-fibrotic activity. In contrast, a significantly higher CR8 concentration is required in primary hepatocytes (right) to induce relevant DNA damage and a comparable apoptosis as in HSCs. Green vertical arrows: strong induction; green angled arrows: moderate induction; red vertical arrows: strong inhibition; red angled arrows: moderate inhibition. (**e**) Graphic conclusion of the study. Altogether murine primary hepatocytes tolerate substantially higher (i.e., 1000 nM) doses of CR8 than primary HSCs (i.e., 100 nM) without prominent cytotoxic effects.

We next tested the impact of CR8 for cell cycle activity of primary murine hepatocytes. Ki-67 stainings revealed that CR8 concentrations ≥500 nM resulted in significant reduction of total cell cycle activity (Figure 6e,f). However, S-phase activity was only significantly reduced by using a concentration of ≥ 5000 nM (Figure 6g,h). Finally, we investigated the effects of CR8 on DNA integrity of primary hepatocytes. Importantly, CR8 only induced DSBs from a high concentration of ≥1000 nM as determined by pH2Ax stainings (Figure 7a,b). As already found in primary HSCs, CR8 did not induce RAD21 or MSH2 gene expression, but also triggered significant down-regulations of both genes at high dosage (≥1000 nM, Figure 7c). Altogether, our data suggest that CR8 has a much less severe impact on DNA integrity in hepatocytes compared to HSCs.

In summary, our study demonstrates that CR8 impairs survival and triggers anti-fibrotic effects in all kinds of HSCs, whereas the effects on hepatocytes are rather moderate (Figure 7d). As a consequence, hepatocytes tolerate much higher (i.e., 1000 nM) CR8 dosages than HSCs (100 nM), before critical effects on survival and cell cycle arrest are observed (Figure 7e).

## 3. Discussion

Liver fibrosis is a wound healing process in response to chronic liver injury, characterized by regenerative proliferation of hepatocytes and the accumulation of extracellular collagen produced by Hepatic Stellate Cells (HSCs) [4]. In extensive previous work we have observed that liver fibrogenesis and fibrosis progression is associated with hepatic cell cycle activity at least in hepatocytes and HSCs [3,7]. Importantly, the E-type cyclin, CcnE1 is of particular importance for liver fibrogenesis, and therapeutic interventional targeting of CcnE1 using RNA interference was shown to mediate a strong anti-fibrotic effect [3]. Yet, it is not known so far, if the anti-fibrotic properties of CcnE1 are dependent on its canonical kinase subunit Cdk2 or related kinases such as Cdk1. This knowledge would be of strong importance as pharmacological small molecules with high specificity for Cdk2 and Cdk1 are already available [22]. For instance, the first generation small-molecular Cdk inhibitor (R)-Roscovitine (also referred to as Seliciclib or CYC202) has been described more than 20 years ago [15] and was shown to inhibit predominantly Cdk2 and Cdk1, but also Cdk5, Cdk7 and Cdk9 although with much lower affinity. However, despite several clinical trials so far [22], Seliciclib has not yet been established in clinical practice, and the efficacy of this drug for treatment of liver diseases with involved cell cycle activity (i.e., fibrosis, hepatocellular carcinoma) is still poorly explored. Recently, a strongly improved second generation analogue of Roscovitine has been developed referred to as CR8. CR8 was shown to target similar Cdks as Roscovitine including Cdk1, Cdk2, Cdk5, Cdk7 and Cdk9, but with much stronger efficacy and a predicted improved anti-tumor potential. Consistently, the ability of CR8 to block cell cycle activity or to induce apoptosis is much higher and reported to be 50-100fold more potent as reported for various cell lines [14,16,23,24]. Thus, CR8 is an improved pan-Cdk inhibitor with promising properties in the treatment of traumatic brain injury, neuroblastoma, chronic lymphocytic leukemia or autosomal dominant polycystic kidney disease as shown recently [23,25,26,27]. However, the anti-proliferative and pro-apoptotic efficiency of CR8 in liver cells has not yet been tested.

For the present study, we wanted to test the hypothesis that the pro-fibrotic function of cyclin E1 is dependent on a cyclin/Cdk kinase activity. Accordingly, inhibition of Cdks, which are capable of forming active kinase complexes with CcnE1 (i.e., Cdk2, Cdk1 [28]) could be a promising therapeutic approach for prospective anti-fibrotic therapies. Thus, the overall aim of this study was to stepwise evaluate potential therapy options for the treatment of liver fibrosis by pharmacological inhibition of Cdks through CR8. To reach this goal, the influence of CR8 on HSC cell lines, primary murine HSCs as well on primary murine hepatocytes was compared in vitro. It has to be emphasized that a successful systemic anti-fibrotic treatment using pan-Cdk inhibitors would require no or at best minor anti-proliferative effects on hepatocytes to maintain full regenerative capacity of the liver, whereas the drug should largely block proliferation of activated hepatic stellate cells, which are the main source of the scar-inducing extracellular matrix. Previous studies using genetic approaches in mice indicated that neither loss of Cdk2 nor Cdk1 had a major effect on liver regeneration at least after partial hepatectomy [29,30].

Our study revealed several promising results. First, CR8 inhibited cell cycle progression and induced cell death in two established, immortalized hepatic stellate cell lines of murine and human origin, respectively. Second, we provided evidence that CR8 also had strong anti-fibrotic effects on primary murine HSC, which much better reflects the in vivo relevance. Finally, we could show that the same CR8 dosages sufficient for blocking pro-fibrotic events in HSCs had at best moderate effects on DNA replication, DNA integrity and survival of primary hepatocytes.

Mechanistically, our analysis implicated that CR8 treatment leads to accumulation of both polyploid DNA (as shown by FACS analysis) and DSBs (indicated by increased H2Ax phosphorylation) in HSCs, probably due to the induction of aberrant replication initiation (re-replication) as suggested previously. DNA damage in turn causes an arrest at the G2- phase (demonstrated by simultaneous increase of cellular DNA content and decrease of histone H3 phosphorylation) and the initiation of intrinsic apoptosis, which is in good agreement with the current literature [20,31]. It should be noted that the accumulation of DSBs in HSCs was not associated with an induction but instead with a down-regulation of DNA repair genes such as RAD21 (involved in DSB-repair) [20,32]) or MSH2 (a central gene during mismatch repair [21]). When interpreting our data, one should take into account that CR8 inhibits—besides Cdk2 and Cdk1—also other Cdks such as Cdk7 and Cdk9, which are involved in general transcription [14,33]. It is therefore possible that the observed down-regulation of RAD21 and MSH2 might be due to inhibitory effects of CR8 on Cdk7 and/or Cdk9. In the same line this could also explain reduced expression of our loading control β-Actin in some GRX cells after treatment with high (i.e., ≥500 nM) concentrations of CR8.

By determination of Rb phosphorylation, we demonstrated efficient inhibition of Cdk activity by CR8 in a dose-dependent manner. It is well established that Cdk2- and Cdk1-dependent phosphorylation of Rb results in its inactivation and subsequent initiation of G1-phase to the S-phase and M-phase transition of the cell cycle [34,35]. In good agreement with our starting hypothesis, inhibition of Cdk activity in HSCs (monitored by reduced Rb phosphorylation) was associated with decreased S-phase and overall reduced cell cycle activity. As already discussed above, the decreased amount of newly replicated DNA was not segregated into daughter cells during mitosis, but accumulated in the G2-Phase of the cell cycle with a significant amount of DSBs giving rise to caspase-3 dependent apoptosis.

In addition to the observed effects of CR8-treatment on HSC proliferation and survival, we also addressed the impact of CR8-mediated Cdk-inhibition on HSC activation and expression of pro-fibrotic markers. Importantly, expression of the intracellular marker αSMA and, to a lesser extent, of the ECM protein Col1A1 [4], were decreased in a dosage-depended manner. It is therefore concluded that CR8 mediates anti-fibrotic effects in HSCs. It is still unclear whether Cdk-inhibition directly restrains the pro-fibrotic properties of HSCs, or if this might be a secondary effect due to the block of proliferation and induction of apoptosis. Alternatively, the anti-fibrotic effects of CR8 could also be explained by off-target inhibition of e.g., Cdk7 and Cdk9, which are involved in general transcription as already stated above [14,33]. Taken together, CR8 acts anti-fibrotic in HSC cell lines through several mechanisms involving cell cycle arrest, apoptosis induction and down-regulation of pro-fibrotic genes.

Our study suggests that CR8 would act anti-fibrotic in both mice and man. In fact, similar anti-fibrotic effects of CR8 were found on murine GRX cells and in human LX-2 cells. However, human LX-2 cells turned out to be approximately five times more sensitive towards CR8 compared to murine GRX cells. It remains to be clarified if these differences regarding sensitivity towards CR8 are true species-dependent disparities or simply variations of two cell lines due to different etiologies.

We have previously shown that primary murine HSCs with a genetic deletion of cyclinE1 show cell cycle arrest, frequent cell death and diminished expression of pro-fibrotic markers after plating indicating that CcnE1 is essential for activation, differentiation and survival of HSCs [7]. In good agreement, knock down of CcnE1 in vivo using small inhibitory RNA turned out to target predominantly HSCs and hepatocytes and was capable of preventing fibrosis progression in an interventional approach in mice [3]. It is obvious that the effect of CR8 on primary HSCs was very similar to our observations in HSCs with genetic deletion of CcnE1. It is therefore tempting to speculate that treatment of fibrotic mice with CR8 could have similar beneficial effects as found after treatment with anti-CcnE1 siRNA. However, experimental proof of this hypothesis was not within the scope of the present study and will be addressed in future work.

An essential condition for using CR8 (and related drug compounds) as a medication for liver fibrosis would be a predominant specificity for HSCs without affecting proliferation and survival of hepatocytes. As outlined before, the regeneration capacity of hepatocytes is absolutely essential in patients with liver fibrosis and underlying chronic hepatitis to preserve residual liver function [36]. Accordingly, we investigated the consequences of CR8-treatment on murine primary hepatocytes. Overall, hepatocytes revealed a much lower sensitivity against CR8 than primary HSCs or HSC lines. Thus, we could identify CR8 concentrations capable of inhibiting the pro-fibrotic properties of CR8 without affecting DNA replication, DNA integrity or survival of hepatocytes. One reason for enhanced tolerance of hepatocytes against CR8 could be the detoxification activity of these cells. In line with this assumption, it has been already shown that (R)-Roscovitine is metabolized in hepatocytes via the cytochrome P450 system and by glucuronidation in vivo [37].

The key findings of our study are summarized in Figure 7d,e: CR8 treatment in both immortalized HSC lines and primary HSCs resulted in cell cycle arrest and apoptosis due to inappropriate accumulation of DNA damage, and in down-regulation of pro-fibrotic genes (Figure 7d, left panel). In contrast, CR8 induces at best minor DNA damage and little apoptosis in primary hepatocytes at concentrations already sufficient for blocking all pro-fibrotic activity in HSCs (Figure 7d, right panel). More precisely, murine primary hepatocytes tolerate substantially higher (i.e., 1000 nM) doses of CR8 than primary HSCs (i.e., 100 nM) or HSC lines (i.e., 500 nM) without prominent cytotoxic effects (Figure 7e). The data of this present proof-of-concept study confirm that pharmacological Cdk-inhibition restricts the pro-fibrotic properties of HSCs, while preserving regeneration capacity of hepatocytes under the same conditions. Therefore, pharmacological Cdk-inhibitors such as CR8 might be promising therapeutic agents for the treatment of liver fibrosis in the future.

## 4. Materials and Methods

### 4.1. Cell Culture Procedures

The origin and cultivation of murine GRX and human LX2 stellate cell lines have been described elsewhere [5,38]. Primary hepatocytes were isolated from 6-10 week old C57BL/6 wildtype mice as described recently [39].

Primary murine HSCs were prepared by collagenase D (Hoffmann-La Roche, Grenzach, Germany)/pronase (Sigma-Aldrich, St. Louis, MS, USA) digestion of livers from 20–30 week old C57BL/6 wildtype mice and subsequently fractionated on density gradients as described before [40]. Cells were cultured on six-well plates (Sarstedt, Nümbrecht, Germany) a density of 0.5 × 10^5^ cells per well in a total volume of 3 mL DMEM (PAN-Biotech, Aidenbach, Germany).

Quantification and viability of all cells was evaluated using a Neubauer counting chamber and trypan blue (1:50, Gibco Thermo Fisher Scientific, Waltham, MA, USA). All cells were cultured at 37 °C in a humidified atmosphere with 5% CO_2_. (S)-CR8 was obtained from Enzo Life Sciences (Farmingdale, NY, USA) and dissolved in DMSO (Sigma-Aldrich) to a stock concentration of 10 mM. Brightfield microscopy was performed using an Axio Vert.A1 microscope with ZEN 2 (blue edition) Software (Carl Zeiss, Oberkochen, Germany).

### 4.2. Measurement of Aminotransferase and Glutamate Dehydrogenase Activity

Alanine aminotransferase (ALT), aspartate aminotransferase (AST) and glutamate dehydrogenase (GLDH) activities were measured in supernatants from cultured primary hepatocytes according to standard methods (UV test at 37  °C) using a Roche Modular preanalytics system (Hoffmann-La Roche).

### 4.3. Quantitative Real-Time PCR (qPCR)

Isolation of total RNA and reverse transcription into cDNA was performed as described recently [3]. Relative quantitative gene expression was measured via real-time PCR using a QuantStudio 6 Flex Real-Time PCR System with QuantStudio Real-Time PCR Software v1.3 (Applied Biosystems, Foster City, CA, USA) Target gene expression was normalized to glyceraldehyde-3-phosphate dehydrogenase (GAPDH) expression as internal standard and calculated as fold induction in comparison to untreated controls. All primer sequences used for qPCR are given in Table A1.

### 4.4. BrdU Incorporation Assay

Activity of DNA-synthesis in the S-phase of the cell cycle was investigated by incorporation of the pyrimidine analog 5-bromo-2′-deoxyuridine (BrdU, AppliChem Biochemica, Darmstadt, Germany, 6 mg/mL in 1× PBS, PAN-Biotech) by stimulation of HSC cell lines and primary hepatocytes two hours bevor harvesting. BrdU incorporation was analyzed by immunofluorescence staining using an antibody specific against BrdU (Santa Cruz Biotechnology, Dallas, TX, USA).

### 4.5. Histology and Immunoblot Analysis

Immunohistochemistry and isolation of whole cell extracts and western blot analysis were performed as described previously [30,41,42]. Antibodies used for probing are listed Table A2. As secondary antibodies, mouse anti-rabbit IgG-HRP (Santa Cruz Biotechnology), m-IgGκ BP-HRP (Santa Cruz Biotechnology), Alexa Fluor 488 goat anti-mouse, Alexa Fluor 488 goat anti-rabbit, Alexa Fluor 594 donkey anti-rabbit (1:200–1:500, Invitrogen Thermo Fisher Scientific, Waltham, MA, USA) were used. For immunoblot analysis, determination of ß-Actin (Abcam, Cambridge, United Kingdom) expression was used as loading control. Immunoblots were visualized by ECL Prime Western Blotting System RPN2232 using an ImageQuant LAS 4000 System with ImageQuant LAS 4000 v1.2 Control Software (GE Healthcare, Chicago, IL, USA), according to the manufacturers protocol. Stained microscopic images were acquired at magnifications of ×200 with a Zeiss Axio Imager.Z1 microscope and AxioVision SE64 Rel. 4.9.1 software (Carl Zeiss).

### 4.6. Determination of Caspase-3 Activity

Apoptosis was determined by quantification of specific caspase-3 enzyme activity (fluorescence units/µg protein/h) in protein lysates from cultured cells as described recently [43]. Briefly, protein lysates were incubated with the artificial substrate AFC-DEVD (Enzo Life Sciences) and the caspase-3 mediated release of AFC from DEVD was quantified by UV spectrometry. Caspase-3 activity was normalized by the used protein concentration and calculated as fold induction in comparison to untreated controls.

### 4.7. Fluorescence Activated Cell Sorting (FACS)

Cultured cells from HSC cell lines were detached by using Trypsin (PAN Biotech) at 37 °C, whereas primary murine HSCs were detached using 500 µL Accutase (Sigma-Aldrich) at room temperature (RT). Cells were pelleted by centrifugation (1200 rpm, 4 °C, 10 min), subsequently fixed with 4% formaldehyde for 15 min at RT, washed with 1x PBS and additional centrifugation. Cell permeabilization was performed using the eBioscience Foxp3/Transcription Factor Staining Buffer Set (Invitrogen Thermo Fisher Scientific) according to the manufacturer’s protocol. For staining of BrdU, the cells were treated with in 100 µL DNAse Solution (1 mg/mL DNAse I, Hoffmann-La Roche, in 1x PBS) and incubated for 1 h at 37 °C. Co-staining of cells was performed using a mix of fluorescence-labeled antibodies against pH3-AL647 (1:50, Cell Signalling Technology, Danver, MA, USA) and cleaved PARP-PE (1:50, BD Biosciences, San Jose, CA, USA) diluted in FACS-blocking buffer (mixture of 0.66% human/rabbit/mouse serum, Sigma-Aldrich, and 1% Bovine Serum Albumin, Sigma-Aldrich in 1× PBS) for 30 min at 4 °C. Antibody details are listed in Table A2. The cellular DNA content was determined by DAPI staining (BD Pharmingen, San Jose, CA, USA, 1:1000 in 1× PBS). Compensation of each fluorochrome was automatically performed using OneComp ebeads (Invitrogen Thermo Fisher Scientific) according to manufacturer’s recommendations. Measurements were performed using a BD FACS Canto (BD Bioscience) and BD LSRFortessa (BD Bioscience) and data were analyzed using FlowJo 7.5 Software (Tree Star, Ashland, OR, USA).

### 4.8. Statistical Analysis

Statistical significance was determined using Graph Pad Prism 7 (GraphPad Software, San Diego, CA, USA). Data were assumed to be normal distributed and analyzed via one-way analysis of variance (ANOVA) with Dunnett’s multiple comparison test, comparing each condition of CR8 treatment to control conditions (0 nM, DMSO). All data are presented as mean ± SD. Significances were defined as *: *p* < 0.05; **: *p* < 0.01; ***: *p* < 0.001; ****: *p* < 0.0001.

## Abbreviations

αSMA	Alpha-smooth-muscle actin
ALT	alanine aminotransferase
AST	aspartate aminotransferase
ATP	Adenosine triphosphate
BrdU	5-bromo-2′-deoxyuridine
CCl_4_	Carbon tetrachloride
Ccn	Cyclin
CcnE1	Cyclin E1
CcnE2	Cyclin E2
Cdk	Cyclin-dependent kinase
Col1A1	Collagen type 1 alpha chain 1
DAPI	4′,6-diamidino-2-phenylindole
DMSO	Dimethyl sulfoxide
DSBs	Double-strand breaks
ECM	Extracellular matrix
FACS	Fluorescence-associated cell sorting, flow cytometry
GAPDH	Glyceraldehyde-3-phosphate dehydrogenase
GLDH	Glutamate dehydrogenase
HSCs	Hepatic Stellate Cells
h	Hours
i.e.,	id est
MSH2	MutS homolog 2
nM	nanomol
PARP-1	Poly (ADP-ribose)-polymerase 1
pH3	phospho-histone H3
pH2Ax	phosphorylated histone H2Ax
Rb	Retinoblastoma protein
siRNA	Small interfering Ribonucleic acid

## Appendix A

**Table A1:** Primer sequences for quantitative real-time PCR.

Gene	Origin	Orientation	Sequence in 5′-3′Orientation
αSMA	murine	sense	TGACAGAGCCACCACTGAACC
		antisense	TCCAGAGTCCAGCACAATACCAGT
	human	sense	CAGCCAAGCACTGTC
		antisense	CCCACCATCACCCCCTGA
Col1A1	murine	sense	TCTGACTGGAAGAGCGGAGAG
		antisense	GGCACAGACGGCTGAGTAGG
	human	sense	GGAATGAAGGGACACAGAGGTT
		antisense	AGTAGCACCATCATTTCCACGA
GAPDH	murine	sense	TGTTGAAGTCACAGGAGACAACCT
		antisense	AACCTGCCAAGTATGATGACATCA
	human	sense	TGTTGAAGTCAGAGGAGACCACCT
		antisense	AACCTGCCAAATATGATGACATCA
MSH2	murine	sense	TCCATCCTCAGGTCAGCAAC
		antisense	TGGGTGGCAAACATGCAAAA
RAD21	murine	sense	GCCGAGATCCAGGTTTCTTC
		antisense	ACATGGGCTTTGGTTAGCTTC

**Table A2:** Antibodies for western blot, immunofluorescence and FACS.

Product	Company
**Primary antibodies (non-conjugated)**	
αSMA [1A4], A2547	Sigma-Aldrich
ß-Actin, A2066	Sigma-Aldrich
BrdU [IIB5], sc-32323	Santa Cruz Biotechnology
Ki-67 [SP6], ab16667	Abcam
Phospho-Histone H2A.X, Ser139 (20E3), #9718	Cell Signaling Technology
Phospho-Rb, Ser807/811, #9308	Cell Signaling Technology
**Primary antibodies (fluorescence-labeled)**	
PE Mouse Anti-Cleaved PARP [F21-852], Asp214	BD Bioscience
Phospho-Histone H3, Ser10 (D2C8) XP, Alexa Fluor 647 Conjugate, #3458	Cell Signaling Technology
**Secondary antibodies (HRP-conjugated)**	
mouse anti-rabbit IgG-HRP, sc-2357	Santa Cruz Biotechnology
m-IgGκ BP-HRP, sc-516102	Santa Cruz Biotechnology
**Secondary antibodies (fluorescence-labeled)**	
Alexa Fluor 488 goat anti-mouse IgG, A-11029	Invitrogen
Alexa Fluor 488 goat anti-rabbit IgG, A-11008	Invitrogen
Alexa Fluor 594 donkey anti-rabbit IgG, A-21207	Invitrogen
